# A Case of Recurrent Hemorrhagic Corpus Luteum with Elevated Follicle-Stimulating Hormone, Controlled by Estrogen/Gestagen Therapy

**DOI:** 10.1155/2020/4098085

**Published:** 2020-07-25

**Authors:** Yoshihide Inayama, Koji Yamanoi, Baku Nakakita, Shimpei Shitanaka, Jumpei Ogura, Tsutomu Ohara, Mie Sakai, Haruka Suzuki, Koji Yasumoto, Ichiro Kishimoto, Yusuke Sagae, Yoshimi Kitawaki, Koh Suginami

**Affiliations:** ^1^Department of Obstetrics and Gynecology, Toyooka Public Hospital, Hyogo, Japan; ^2^Department of Endocrinology and Diabetes, Toyooka Public Hospital, Hyogo, Japan; ^3^Department of Gynecology and Obstetrics, Kyoto University Graduate School of Medicine, Kyoto 606-8507, Japan

## Abstract

A high secretion of follicle-stimulating hormone (FSH) in reproductive-aged women is unusual. We report a case of recurrent corpus luteum hemorrhage and subsequent ovarian torsion with markedly elevated FSH levels in a reproductive-aged woman in the absence of functional gonadotroph adenoma (FGA) or premature ovarian failure (POF). A 22-year-old nulligravid woman with a history of bilateral hemorrhagic corpus luteum and subsequent ovarian torsion presented with acute abdominal pain. An emergency salpingo-oophorectomy of the right side was performed, and the right ovarian torsion due to hemorrhagic corpus luteum was diagnosed. Laboratory tests revealed markedly elevated FSH levels (77.6 mIU/mL). FGA was suspected, but no evidence of tumor was identified. The left ovary enlarged again at one-month follow-up. Estrogen/gestagen therapy (EGT) was started, which reduced the enlarged ovary to normal size. Two years later, her pituitary hormonal status was evaluated in detail. Besides markedly elevated FSH level, slightly elevated LH (31.2 mIU/mL), normal total inhibin B (35.3 pg/ml), abnormally low anti-Müllerian hormone (AMH) (<0.03 ng/mL), and poor FSH response to gonadotropin-releasing hormone stimulation test were found. In the absence of FGA, we conclude that certain disorders of inhibitory factors for FSH function, including inhibin and AMH may exist, which could attribute to the patient's symptoms. EGT was very effective in suppressing the ovarian hyperactivity.

## 1. Introduction

High levels of follicle-stimulating hormone (FSH) in a reproductive age woman is unusual. Functional gonadotroph adenoma (FGA) and premature ovarian failure (POF) are known to be the primary causes for this condition. Patients with FGA occasionally develop ovarian hyperstimulation syndrome (OHSS) [1]. However, there have been no reports of enlarged ovaries with elevated FSH levels in the absence of FGA in reproductive-aged women.

Here, we report a case of recurrent ovarian enlargement followed by ovarian torsion due to hemorrhagic corpus luteum in a nulligravid, reproductive-aged woman. Laboratory investigations revealed markedly elevated FSH levels, but FGA was not identified. To our knowledge, this is the first case of recurrent hemorrhagic corpus luteum in a nulligravid, reproductive-aged woman without FGA.

## 2. Case Presentation

### 2.1. Recurrence of Ovarian Enlargement and Torsion due to Hemorrhagic Corpus Luteum

**Figure 1:**
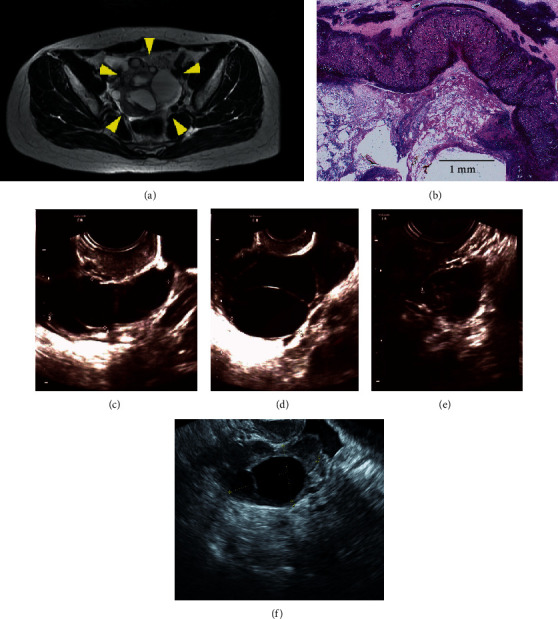
(a) Pelvic magnetic resonance imaging at diagnosis of ovarian torsion (T2-weighted axial image). The left ovary is indicated by the yellow allows. (b) Magnification (×40) of hematoxylin and eosin staining of the resected ovary. Scale is shown in the figure. Hemorrhagic corpus luteum is observed. (c) The left ovary at the one-month follow-up evaluation after emergent right salpingo-oophorectomy. The ovary is enlarged, measuring 4.4 × 2.4 cm. (d) The left ovary before the start of estrogen/gestagen therapy. The ovary is enlarged measuring 7.5 × 5.5 cm. (e) The left ovary five months after the start of estrogen/gestagen therapy. The ovary is reduced to normal size (3.9 × 2.0 cm). (f) The left ovary during estrogen/gestagen therapy. The ovary remained at a normal size (3.5 × 2.1 cm).

A 22-year-old nulligravid woman presented with acute abdominal pain. Her weight and height were 53 kg and 150 cm. Her menstruation started at 10 years of age and has been regular. She had a history of enlargement of bilateral ovaries and left ovarian torsion 3 years ago, for which she underwent laparoscopic ovarian detorsion and bilateral cystectomy. Pathological examination documented bilateral hemorrhagic corpus luteum. At presentation, ultrasonography and pelvic magnetic resonance imaging (MRI) revealed enlarged right ovary with multiple cysts (up to 7 cm in diameter). Ovarian torsion was highly suspected (Figure 1(a)). An emergency laparotomy was conducted, which revealed a dark red enlarged right ovary that had twisted 540°. Detorsion of the right ovary was performed, but it was determined to be too necrotic to be preserved. Therefore, a right salpingo-oophorectomy was performed. Hemorrhagic corpus luteum was pathologically confirmed (Figure 1(b)).

### 2.2. Start of Estrogen/Gestagen Therapy (EGT)

The patient's menstruation resumed within 1 month after surgery. At the one-month follow-up, the left ovary was enlarged again (4.4 × 2.4 cm, Figure 1(c)). Blood examination on cycle date (CD) 9 revealed a markedly elevated FSH level of 77.6 mIU/mL and a slightly elevated luteinizing hormone (LH) level of 54.7 mIU/m. Estradiol level was 78.3 pg/ml (Table 1). Polycystic ovarian syndrome (PCOS) was not suspected because her menstruation was regular and the LH was not dominant. Moreover, the polycystic ovary seen in this patient was not similar to polycystic ovaries typically seen in PCOS. FGA was suspected, but a pituitary MRI (1.5T) showed no evidence of tumor. To control enlargement of the ovaries, we started the patient on EGT, which consisted of conjugated estrogens 0.625 mg per day for 10 days followed by conjugated estrogens 0.625 mg and chlormadinone acetate 2 mg for the next 10 days. After a few cycles of EGT, the enlarged ovary reduced from 7.5 × 5.5 cm (Figure 1(d)) to normal size (five months after initiation of EGT, 3.9 × 2.0 cm, Figure 1(e)). Her estrogen level increased up to 139.4 pg/ml three months after the initiation of EGT. EGT was continued for over two years without any significant side-effects. Regular withdrawal bleeding was observed and the ovary size was essentially stable (Figure 1(f), 3.5 × 2.1 cm).

### 2.3. Detailed Pituitary Hormonal Stress Tests

**Figure 2:**
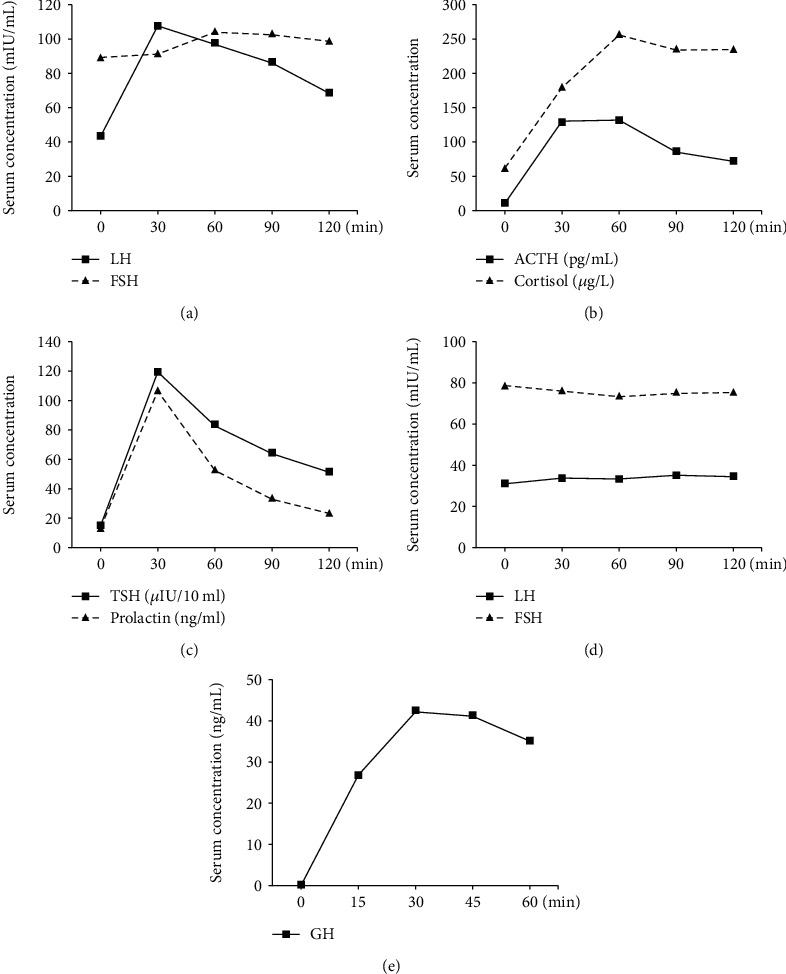
The results of hormone stimulation test. (a) The result of gonadotropin-releasing hormone (GnRH) stimulation test on cycle date (CD) 4. (b) The result of corticotropin-releasing hormone stimulation test on CD5. No abnormal findings were observed. ACTH: aderenocorticotropic hormone. (c, d) The results of thyroid-stimulating hormone test on CD6. No abnormal findings were observed. TSH: thyroid-stimulating hormone. (e) The result of growth hormone releasing peptide-2 stimulation test on CD9. No abnormal findings were observed.

**Figure 3:**
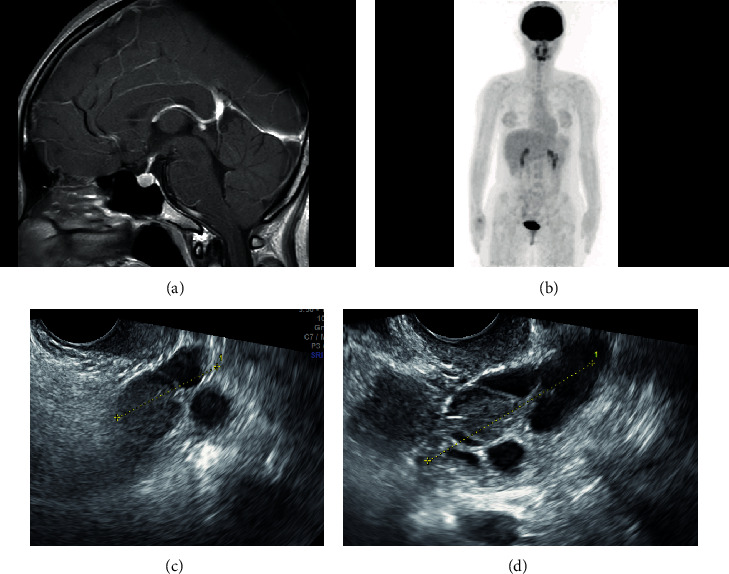
(a) Pituitary magnetic resonance imaging (gadolinium-enhanced T1-weighted sagittal image). No tumor is observed in the pituitary gland. (b) Result of positron emission tomography. No ectopic tumor is observed. (c, d) The left ovary after estrogen/gestagen therapy was discontinued. (c) Cycle date 3: 3.0 cm. (d) Cycle date 9: 4.8 cm.

After EGT was continued for two and a half years, the patient requested for further investigation of her condition to evaluate her fertility. We deduced that she could have disorders in her hormone regulation system, and we planned to check her pituitary hormonal status in more detail in the absence of EGT. The FSH level was still elevated to 82.6 pg/ml and the estradiol level was 82.6 pg/ml just before the EGT was discontinued. Withdrawal bleeding started on the fifth day after the EGT was stopped. Pituitary hormone stimulation tests were conducted during this menstrual cycle as the discontinuation of EGT might result in the recurrence of ovarian enlargement. Gonadotropin-releasing hormone (GnRH) stimulation test on CD4 revealed an elevated FSH level of 89.4 mIU/mL with poor response to GnRH stimulation (maximum, 107.9 mIU/mL) and a slightly elevated LH level of 43.4 mIU/mL (Figure 2(a)). The details of GnRH stimulation test are described in the Supplementary Text. No abnormal findings were observed in corticotropin-releasing hormone stimulation test (Figure 2(b)), thyroid-stimulating hormone test (Figures 2(c) and 2(d)), and growth-hormone-releasing peptide-2 stimulation test (Figure 2(e)). Contrast-enhanced pituitary MRI (3T) and ^18^F-fluorodeoxyglucose positron emission tomography (FDG-PET) were conducted, but no evidence of pituitary adenoma or ectopic tumor was identified (Figures 3(a) and 3(b)).

Next, we investigated inhibin and anti-Müllerian hormone (AMH) levels because they are strongly related to FSH unction. Methods of evaluating inhibin level are described in Supplementary Text. The total inhibin level was 156.8 pg/mL, and inhibin B level was 35.3 pg/mL (Table 1). On the other hand, the AMH level was remarkably low at <0.03 ng/mL (Table 1). Though the left ovary became slightly enlarged during the treatment-free period (CD3: 3.0 cm, Figure 3(c); CD9: 4.8 cm, Figure 3(d)), it reduced to normal size after the treatment was resumed.

## 3. Discussion

We documented a rare case of recurrent ovarian enlargement followed by ovarian torsion due to hemorrhagic corpus luteum. EGT was effective in preventing further ovarian enlargement in this patient. Interestingly, in some cases of FGA with OHSS, hormone therapy was reported to be useful [2, 3]. It has been argued that exogenous estrogen might have provided negative feedback on FSH secretion and hence prevented ovarian hyperstimulation. In our case, however, FSH secretion remained elevated during EGT. We speculate that EGT may suppress the pulsatile secretion of GnRH, resulting in inhibition of ovarian stimulation. Another possibility is that the exogenous estrogen dose may have been sufficient to reduce the size of the ovary but not enough to suppress the FSH secretion and serum levels significantly. Although the underlying etiology remains unclear, EGT might be considered as a treatment option to control ovarian stimulation in patients with FGA or HL, who have symptoms similar to our patient.

Despite detailed hormonal evaluation, we could not elucidate the exact pathophysiology of this condition. However, we can speculate certain possibilities, based on our results.

First, this case was characterized by markedly elevated FSH levels. In a reproductive-aged woman, POF or FGA may be the primary cause for this condition [1]. We think that POF is unlikely in this patient although she did present with a low AMH level and an elevated FSH level. Firstly, serum inhibin B usually declines in the early menopausal transition period and its level is low after menopause, but her inhibin B level had not declined [4, 5]. Secondly, although her FSH was kept at an extremely high level for more than two years, the secretion of estradiol was still maintained even when the EGT was not administrated. Moreover, the ovarian enlargement recurred when the EGT was discontinued two years later. We therefore suspected the presence of FGA. However, no evidence of tumor was documented by MRI or FDG-PET. As small pituitary tumors are not always identified by MRI [6], hormonal assays were conducted. While FSH secretion was remarkably elevated and LH secretion was slightly elevated, prolactin and estrogen levels were within normal range. We reviewed some case reports of FGA (Supplementary Text, Supplementary Figure S1, Supplementary Figure S2, Supplementary Table S1), and our results were in contrast with reported cases of FGA, which were characterized by slightly elevated FSH, decreased LH, and elevated prolactin and estrogen secretion (Table 1). Moreover, the results of GnRH stimulation tests were not comparable to the same in women with FGA (Supplementary Table S2) Pathological examination revealed that the ovaries were enlarged with hemorrhagic corpora lutea, the so-called hyperreactio luteinalis (HL). On the other hand, FGA patients with enlarged ovaries usually have associated ascites, a condition similar to OHSS [1]. All these data suggest that the etiology of hormonal imbalance in our patient differs from that in patients with FGA.

Besides markedly elevated FSH levels, inhibin levels also seem to be a unique characteristic. Inhibin plays an important role in the negative regulation of FSH secretion. In some cases of FGA, inhibin levels are elevated (Table 1), which is probably caused by elevated FSH. On the other hand, the total inhibin and inhibin B levels remained relatively low in our case. This might indicate that the negative feedback by inhibin may not have been functioning effectively. We suspect that in our case certain disorders involving the function of inhibin may exist, resulting in an abnormal secretion of FSH. Further analysis regarding protein structure and mutation of inhibin might help us to elucidate the exact pathophysiology. In addition, certain disorders involving the feedback mechanisms to the hypothalamus may also exist. Further investigation is needed to elucidate the detailed mechanisms in our case.

While the inhibin B level remained within normal range, the AMH level was abnormally low. AMH and inhibin B are both known as biomarkers to assess ovarian reserve [7]. This conflicting result suggests that the condition might be complicated with AMH deficiency. AMH deficiency can cause genital abnormalities in a 46XY genetic male, while it is considered to be clinically insignificant in a 46XX genetic female [8]. However, in a mouse model, FSH secretion is affected and more follicles are recruited when AMH is knocked-out [9]. It is possible that enhanced FSH secretion caused by AMH deficiency might have exaggerated the clinical course of this patient.

Chromosomal abnormalities are also potential causes of our patient's condition. For example, Turner syndrome, especially Turner mosaicism, is sometimes known to induce elevated levels of FSH and hyperstimulation of follicles can occur in Turner mosaicism [10]. Chromosomal analysis can be considered if the patient agrees.

In conclusion, we documented a rare case of recurrent hemorrhagic corpus luteum with markedly elevated FSH. EGT was effective to suppress ovarian hyperactivity. We speculate that certain disorders related to inhibitory factors for FSH function including inhibin and AMH may exist in this case. It is obvious that the cause of her condition has not been fully clarified. Further reports in this area are required to address the exact pathophysiology.

## Figures and Tables

**Table 1:** Hormone levels. List of endocrine hormone levels of this patient and reported cases of FGA, which are quoted from mini-review (Supplementary Table S1).

Hormone level	Reference value	Postoperative one month	Postoperative two and half years	Values in reported cases of FGA
LH (mIU/mL)	fp 1.1-12.1, lp 0.7-21.6	54.7	31.2	0.5 [0.1-0.8]
FSH (mIU/mL)	fp 2.6-11.9, lp 1.4-9.6	77.6	62.0	10.3 [8.8-15.9]
Estradiol (pg/mL)	fp < 121.6, lp < 463.4	78.3	82.6	601 [322-1758]
Progesterone (ng/mL)	fp < 1, lp < 15.5	1.18	1.1	2.8 [1.4-4.4]
Total inhibin (pg/mL)		N/A	156.8	
Inhibi B (pg/mL)		N/A	35.3	506 [231-812]
AMH (ng/mL)	2.00-12.5	N/A	<0.03	0.41-1.62
Testosterone (ng/mL)	0.11-0.47	N/A	0.28	
Prolactin (ng/mL)	3.9-31.7	8.13	14.6	57 [32-70]
TSH (*μ*IU/mL)	0.35-4.94	1.07	0.97	
Free T3 (pg/mL)	1.88-3.18	N/A	2.0	
Free T4 (ng/dL)	0.70-1.48	1.7	N/A	
ACTH (pg/mL)	7.2-63.3	25.0	12.4	
Cortisol (*μ*g/dL)	6.2-19.4	6.32	9.43	
GH (ng/mL)	0-0.765	0.29	0.08	
IGF-1 (ng/mL)	151-375	N/A	140	

AMH: anti-Müllerian hormone; FGA: functional gonadotroph adenoma; fp: follicular phase; IGF-1: insulin-like growth factor-1; lp: luteal phase; N/A: not available.

## Supplementary Materials

Supplementary Materials Supplementary Text: method of evaluating inhibin level in serum, method for conducting the mini review, supplementary table legends, and supplementary figure legends. Supplementary Table S1: Hormonal values in the reported cases in the mini-review. Supplementary Table S2: the results of gonadotropin-releasing hormone (GnRH) stimulation test in the present case and mini-review. Supplementary Figure S1: images of pituitary MRI, hematoxylin and eosin staining of the resected ovary, pelvic MRI, and FDG-PET. Supplementary Figure S2: schematic of the method for conducting the mini-review.
