# Supplementary material for: A Case of Recurrent Hemorrhagic Corpus Luteum with Elevated Follicle-Stimulating Hormone, Controlled by Estrogen/Gestagen Therapy
Source: Case Rep Obstet Gynecol. 2020 Jul 25;2020:4098085. doi: 10.1155/2020/4098085 (PMC7399774; doi:10.1155/2020/4098085)
Supplement: Supplementary Materials — Supplementary Text: method of evaluating inhibin level in serum, method for conducting the mini review, supplementary table legends, and supplementary figure legends. Supplementary Table S1: Hormonal values in the reported cases in the mini-review. Supplementary Table S2: the results of gonadotropin-releasing hormone (GnRH) stimulation test in the present case and mini-review. Supplementary Figure S1: images of pituitary MRI, hematoxylin and eosin staining of the resected ovary, pelvic MRI, and FDG-PET. Supplementary Figure S2: schematic of the method for conducting the mini-review. [file 4098085.f1.ppt]

## Slide 1
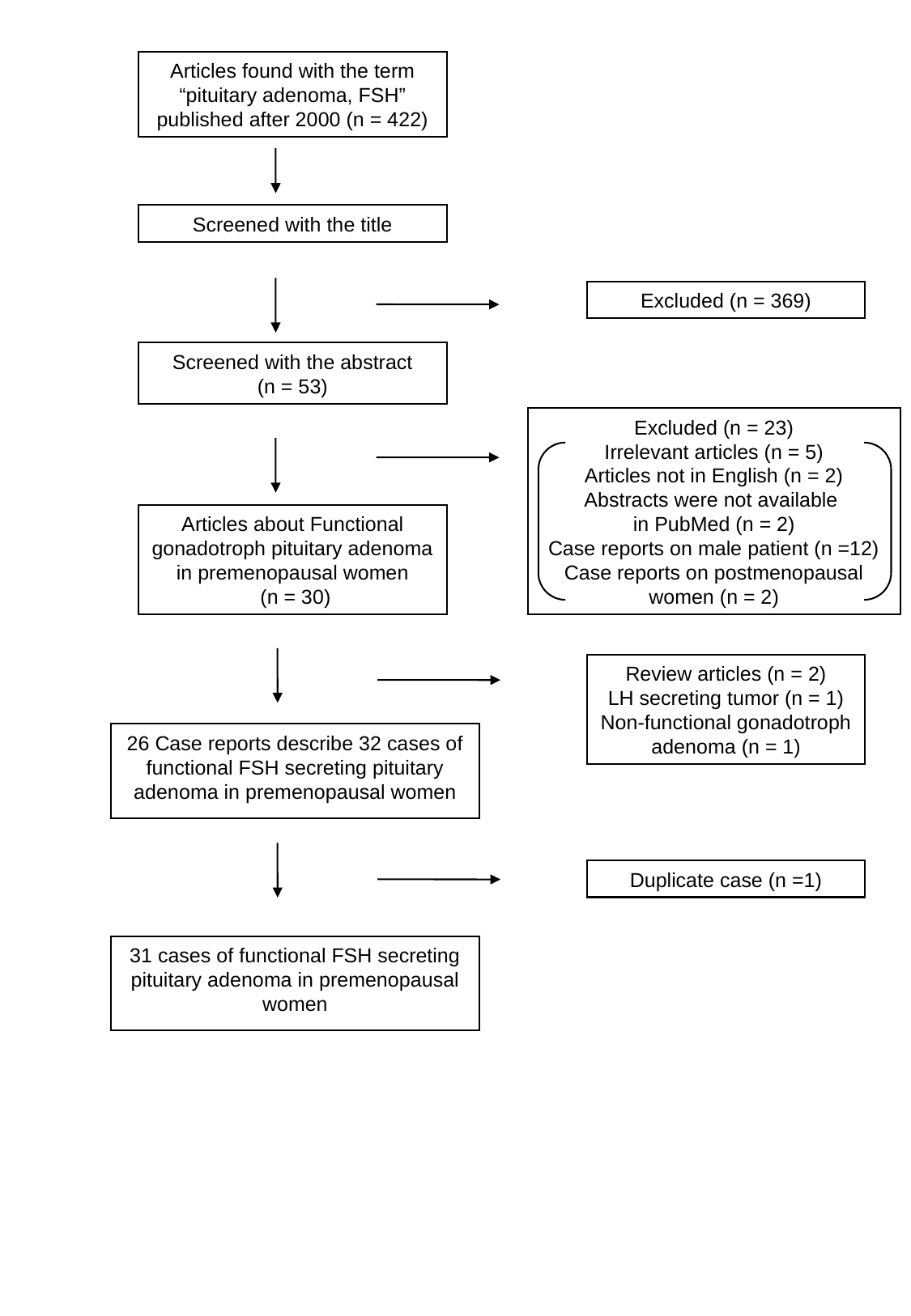

Articles found with the term “pituitary adenoma, FSH” published after 2000 (n = 422)
Screened with the title
Excluded (n = 369)
Screened with the abstract
(n = 53)
Excluded (n = 23)
Irrelevant articles (n = 5)
Articles not in English (n = 2)
Abstracts were not available
in PubMed (n = 2)
Case reports on male patient (n =12)
Case reports on postmenopausal women (n = 2)
Articles about Functional gonadotroph pituitary adenoma in premenopausal women
 (n = 30)
Review articles (n = 2)
LH secreting tumor (n = 1)
Non-functional gonadotroph adenoma (n = 1)
26 Case reports describe 32 cases of functional FSH secreting pituitary adenoma in premenopausal women
Duplicate case (n =1)
31 cases of functional FSH secreting pituitary adenoma in premenopausal women
